# The Contrast Effect in Temporal and Probabilistic Discounting

**DOI:** 10.3389/fpsyg.2016.00304

**Published:** 2016-03-07

**Authors:** Cheng Chen, Guibing He

**Affiliations:** Department of Psychology and Behavioral Sciences, Zhejiang UniversityHangzhou, China

**Keywords:** temporal discounting, probabilistic discounting, contrast effect, psychological distance, decision by sampling

## Abstract

In this information age, messages related to time, and uncertainty surround us. At the same time, our daily lives are filled with decisions accompanied by temporal delay or uncertainty. Will such information influence our temporal and probabilistic discounting? The authors address this question from the perspectives of decision by sampling (DbS) theory and psychological distance theory. Studies 1 and 2 investigated the effect of contextual messages on temporal discounting and probabilistic discounting, respectively. The results indicated that participants who memorized messages about long-term and low-probability events rated delay or uncertainty as mentally closer and exhibited a less degree of value discounting than those who memorized messages regarding short-term and high-probability events. In addition, a sense of distance from present or reality mediated the effect of contextual messages on value discounting. The implications of the current findings for theory and applications are discussed.

## Introduction

We live in an information age. Thanks to scientific progress and the widespread power of the internet, millions of pieces of information regarding time or uncertainty—for example, the self-updating rate of human cells, the half-lives of atoms, the duplication accuracy of DNA, and the success rate of iris certification systems—are accessible to us. At the same time, in our daily lives, we must constantly make decisions under conditions of delay and uncertainty. Given that the outcomes of these decisions occur in the future or with uncertainty, our value estimations of them are somewhat diminished.

Does information pertaining to time or probability, such as the half-lives of atoms and duplication accuracy rate of DNA, influence our assessments of delayed or uncertain outcomes? If so, what is the underlying mechanism? We address these two questions in the current paper.

### Temporal and probabilistic discounting

Temporal and probabilistic discounting refers to the devaluation of delayed or uncertain outcomes from their nominal values (e.g., Green and Myerson, 2004). In view of the significant role of time and uncertainty in our daily lives, it is not surprising that temporal and probabilistic discounting remains a key focus in the decision-making field.

Numerous findings on the topic of time have emerged. It has been shown that people discount future rewards hyperbolically (e.g., Rachlin et al., 1991). That is, while individuals sharply devalue rewards in the near future, they exhibit relatively smooth discounting trends as delays increase. In addition, factors that impact the extent of temporal discounting have been investigated. In particular, attributes of outcomes, such as valence (positive or negative), magnitude (large or small) (e.g., Green and Myerson, 2004), and types of reward (monetary, directly consumable, or environmental; Estle et al., 2007; Hardisty and Weber, 2009), are found to impact people's inter-temporal decisions. Besides these, characteristics of decision makers, such as age (Steinberg et al., 2009), sense of power (Joshi and Fast, 2013), and future-self connectedness (Bartels and Rips, 2010) can also shape the temporal discounting rate. Recently, some scholars approached temporal discounting from the perspective of the subjective perception of time (Zauberman et al., 2009). They revealed that insufficient sensitivity to prospective time duration contributes to hyperbolic discounting and that when people become more sensitive to the time horizon, their hyperbolic discounting decreases.

Parallel findings have been obtained in the field of probabilistic discounting. For instance, it has been verified that hyperbolic models capture probabilistic discounting more effectively than exponential or other models (e.g., Rachlin et al., 1991). At the same time, people discount uncertain outcomes to a greater degree for prospective gains than for prospective losses and for large amounts than for small amounts (e.g., Green and Myerson, 2004). In addition, the role of probability perception has been highlighted. Kahneman and Tversky depicted the relationship between objective probability and subjective weighting in their Prospect Theory (Kahneman and Tversky, 1979). As they argued, the relationship between these two variables is nonlinear. Specifically, individuals tend to overestimate the value of a small probability but underestimate the value of a medium or large probability.

We return to the question raised above, i.e., does information regarding time or probability affect our evaluations of delayed or uncertain outcomes? If so, how does this phenomenon occur? We address this issue from the perspective of subjective perception. That is, information related to time or probability will impact our perceptions of delay and uncertainty, which in turn may affect our temporal and probabilistic discounting.

### The contrast effect

The contrast effect is a contextual effect whereby background stimuli alter one's assessment of target stimuli. In particular, it refers to the subjective differentiation of target stimuli from contextual ones. This effect has been demonstrated in a wide variety of domains, including sensory perception (e.g., Sarris, 1967; Pol et al., 1998), personality trait judgment (e.g., Herr, 1986; Manis et al., 1988), and hedonic evaluation (e.g., Zellner et al., 2003, 2006). For instance, research on sensory perception found that the odor of stimuli was perceived as more intense when preceded by a weak odor stimulus than when preceded by a strong odor one. In the domain of social cognition, the target person was judged as more hostile following a moderate hostility exemplar than an extreme hostility one. Similarly, hedonically neutral target stimuli were rated as worse if preceded by good contextual stimulus and rated as better if preceded by bad contextual one.

This comparison effect can also be observed in decision-making settings. In the decision by sampling (DbS) theory, Stewart et al. (2006) stressed that it should not be assumed that people have stable internal scales along which they represent value, probability, temporal duration, or any other magnitudes and that, instead, people can sample items from both the immediate context and memory and assess the value of a target through comparison. This idea has been verified in several studies. For instance, Ungemach et al. (2011) found that, consistent with the hypothesis of DbS, incidental values of the environment, such as the price on a supermarket receipt, the probability that there will be rain on the following day, and the next birthday date, could influence our preferences for risky and inter-temporal choices.

Based on the above evidence, we expect that the contextual information referring to time or probability will impact temporal and probabilistic discounting through the contrast effect. In particular, a given delay will be perceived as closer when preceded by messages regarding long-time events than when preceded by messages concerning short-time events, leading to a less degree of temporal discounting. Similarly, a given probability will be revised upward when preceded by messages about low-probability events but not when preceded by information about high-probability events, inducing a less degree of probabilistic discounting.

### The inter-domain contrast effect

Of note, the above analysis focuses on time-time and probability-probability contrast effects, while the inter-domain effect remains unknown. What is the impact of time-related information on probabilistic discounting, and vice versa? Will such messages have comparable influences on value estimation across domains?

In the current paper, we apply psychological distance theory (Trope and Liberman, 2010) to determine time-probability inter-domain impacts within the contrast effect. Psychological distance theory consists of two primary hypotheses. The first one is that the manner in which we perceive an object depends on our psychological distance from it. If the object is mentally distant from us, we will focus on its high-level features, which are abstract, primary and goal-relevant. By contrast, if it is psychologically close to us, we will mainly consider its low-level features, i.e., concrete, secondary, and goal-irrelevant ones. The second hypothesis predicts that temporal, probabilistic, social and spatial distances are similar in essence and could be accounted for by one construct—psychological distance.

Both of these hypotheses have been examined in numerous studies. Regarding the first hypothesis, for instance, Liberman and Trope (1998) revealed that participants preferred to describe distant future activities rather than near future activities in high-level terms and gave more consideration to the desirability than the feasibility of actions when making decisions regarding distant future activities. Similarly, a higher level of construal was found to be associated with actions occurring in a distant location (Fujita et al., 2006), unlikely to occur (Wakslak et al., 2006), or implemented by dissimilar others (Liviatan et al., 2008). Concerning the second hypothesis, a study conducted by Bar-Anan et al. (2007) presented participants with a series of words with meanings related to temporal, probabilistic or social distance and asked them to indicate the locations of these words. The results showed that participants responded more rapidly when the meaning of a word matched its location than when it did not. Fiedler et al. (2012) found that people's estimations of temporal, probabilistic, spatial, and personal distances of specific social behaviors were all positively related. In addition, when the distances of these social behaviors were manipulated along two dimensions, participants' judgments of the distances along the remaining two dimensions changed in the same direction.

As time delay and uncertainty are manifestations of psychological distance from present (temporal distance) and from reality (probabilistic distance), respectively, we predict that messages related to time and uncertainty will have parallel effects on value discounting. In particular, messages pertaining to long duration or low probability, both of which are mentally distant, should cause medium temporal delay and uncertainty to be perceived as closer in the mind and lead to a less degree of temporal and probabilistic discounting. At the same time, messages pertaining to short duration or high probability, both of which are psychologically adjacent, should cause medium delay and uncertainty to be perceived as more remote and induce a greater degree of value discounting.

## Study 1: impact of contextual messages on estimation of temporal distance and present value

### Methods

#### Ethics statement

The current study was approved by the Research Ethics Board of Zhejiang University. All participants provided written informed consent before participating in the experiment.

#### Participants

A total of 185 undergraduates (105 female and 80 male) participated in Study 1. Their mean age was 21.4 years (SD = 1.12 years).

#### Design and procedure

Study 1 consisted of a memory task and a decision task and was implemented on the computer. In the memory task, participants were asked to memorize three messages related to time or probability. There were five conditions: long-time condition, short-time condition, low-probability condition, high-probability condition, and control condition^1^ (the materials used in each condition are presented in the Appendix). Participants were randomly assigned to one of these conditions, and the order of messages in each condition was counterbalanced. Those in the control group were not asked to memorize any messages and began with the decision task.

The decision task was composed of two parts: temporal distance estimation and present value assessment. In the temporal distance estimation task, a 180-mm line, labeled “very near” on the left end and “very far” on the right end, was presented on a screen (Zauberman et al., 2009). Participants were asked to adjust a bar placed in the middle of the line to indicate their psychological distance estimation of 3 months from the present day. We recorded the length between the left end of the line and the final location of the bar and performed a linear transformation resulting in a 0–100 scale, with 0 representing “very near” and 100 representing “very far.” The transformed data were used as the index of temporal distance estimation.

In the temporal discounting task, participants were presented with a 180-mm line similar to that in the temporal distance estimation task except that it was labeled “0 RMB” on the left end and “750 RMB” on the right end. They were asked to imagine receiving 750 RMB in 3 months and to adjust the bar to illustrate the present value of that money. The distance from the left end of the line to the final location of the bar was transformed linearly into a 0–750 scale. The transformed data were used to indicate the discounted value.

The order of the temporal distance estimation task and the present value assessment task was counterbalanced.

### Results

#### Effect of contextual message on temporal distance and present value estimation

The means and standard deviations of the temporal distance and present value estimations under each condition are presented in Table 1.

**Table 1:** **Temporal distance and present value estimations under each condition**.

	**Temporal distance**		**Present value**	
	**M**	**SD**	**M**	**SD**
Long-time condition (*n* = 37)	35.78	10.13	450.95	112.03
Short-time condition (*n* = 38)	43.03	12.04	383.82	108.09
Low-probability condition (*n* = 38)	37.39	11.65	439.61	106.86
High-probability condition (*n* = 39)	42.41	11.14	387.95	108.25
Control group (*n* = 33)	39.42	11.03	411.67	102.57

A one-factor ANOVA illustrated that the effect of contextual messages on temporal distance estimation was significant [*F*_(4, 180)_ = 2.93, *p* < 0.05]. Although the differences between the control group and other conditions were not significant, participants in the short-time, and high-probability conditions viewed the 3-month delay as more distant than those in the long-time and low-probability conditions (t_ST−LT_ = 2.79, *p* < 0.01; t_ST−LP_ = 2.19, *p* < 0.05; t_HP−LT_ = 2.57, *p* < 0.05; t_HP−LP_ = 1.96, *p* = 0.05). In addition, there were no significant differences between the long-time and low-probability conditions or between the short-time and high-probability conditions (*p*s > 0.18). Therefore, contextual messages under comparable psychological distance conditions had identical effects on temporal distance estimation.

The effect of contextual messages was also found in the present value estimation task [*F*_(4, 180)_ = 2.94, *p* < 0.05]. Participants who memorized messages about short-time and high-probability events discounted the value of the 750 RMB received after 3 months to a greater extent than those who memorized messages about long-time and low-probability events (t_ST−LT_ = −2.70, *p* < 0.01; t_ST−LP_ = −2.26, *p* < 0.05; t_HP−LT_ = −2.55, *p* < 0.05; t_HP−LP_ = −2.10, *p* < 0.05). No other significant differences were found between the control group and other conditions, between the long-time condition and low-probability condition, or between the short-time condition and high-probability condition (*p*s > 0.13).

#### Mediation effect of psychological distance

**Figure 1:**
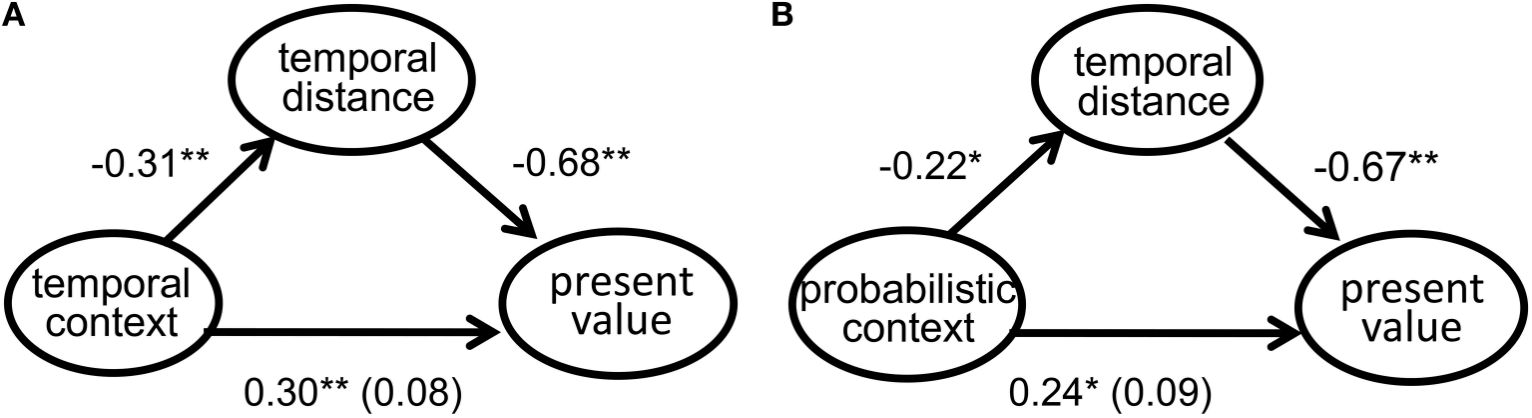
**(A)** Mediation analysis of temporal distance between temporal contexts and present value estimation. **(B)** Mediation analysis of temporal distance between probabilistic contexts and present value estimation.

We assumed that for participants in the long-time condition, a decrease in the magnitude of temporal distance would account for their less degrees of delay discounting. A mediation analysis (Judd and Kenny, 1981; Baron and Kenny, 1986) indicated that types of time-related contextual messages predicted the estimations of both present value (β = 0.30, *p* = 0.01) and temporal distance (β = −0.31, *p* < 0.01) (the long-time condition was coded 1, and the short-time condition was coded 0). When present value assessment was simultaneously predicted by types of temporal contextual messages and temporal distance estimation, the predictive power of the former was no longer significant (β = 0.08, *p* = 0.34), while that of the latter remained significant (β = −0.68, *p* < 0.01; see Figure 1A). Hence, the temporal distance mediated the relationship between types of temporal contextual messages and present value estimation.

Furthermore, a mediating relationship was also found among types of probability-related contextual message, temporal distance estimation, and present value assessment. First, types of probability-related contextual messages affected present value assessment (β = 0.24, *p* < 0.05; the low-probability condition was coded 1, and the high-probability condition was coded 0). Second, types of probability-related contextual messages affected temporal distance estimation (β = −0.22, *p* = 0.05). Third, when present value assessment was regressed on both types of probabilistic contextual messages and temporal distance estimation, only the latter significantly predicted present value (β = 0.09, *p* = 0.30; β = −0.67, *p* < 0.01; see Figure 1B). Therefore, probabilistic context impacted temporal discounting through temporal distance estimation.

## Study 2: impact of contextual messages on estimation of probabilistic distance and certainty equivalence

### Methods

#### Participants

A total of 183 undergraduates (98 female and 85 male) participated in Study 2. Their mean age was 21.8 years (SD = 0.94 years).

#### Design and procedure

Study 2 was composed of a memory task and a decision task. In the memory task, participants were asked to memorize three messages related to time or probability. It included five conditions: long-time condition, short-time condition, low-probability condition, high-probability condition, and control condition. In the decision task, they were asked to indicate the psychological distance of a probability of 72% from certainty and the certainty equivalence of 750 RMB received with that probability. The details of Study 2 are similar to those of Study 1.

### Result

#### Effect of the contextual message on probabilistic distance and certainty equivalence estimations

The means and standard deviations of the probabilistic distance and certainty equivalence estimations under each condition are presented in Table 2.

**Table 2:** **Probabilistic distance and certainty equivalence estimations under each condition**.

	**Probabilistic distance**		**Certainty equivalence**	
	**M**	**SD**	**M**	**SD**
Long-time condition (*n* = 38)	31.47	9.78	456.84	95.72
Short-time condition (*n* = 39)	35.72	10.93	404.87	111.92
Low-probability condition (*n* = 35)	30.00	8.70	473.00	109.47
High-probability condition (*n* = 37)	36.46	11.19	403.24	97.27
Control group (*n* = 34)	33.15	12.07	433.53	105.14

A one-factor ANOVA revealed the effect of contextual messages on probabilistic distance estimation [*F*_(4, 178)_ = 2.46, *p* < 0.05]. Although the control group performed similarly to the other groups, participants in the short-time and high-probability conditions rated the probability of 72% as further from certainty than those in the long-time and low-probability conditions (t_ST−LT_ = 1.76, *p* = 0.08; t_ST−LP_ = 2.32, *p* < 0.05; t_HP−LT_ = 2.04, *p* < 0.05; t_HP−LP_ = 2.59, *p* < 0.05). In addition, there were no differences between the long-time and low-probability groups or between the short-time and high-probability groups (*p*s > 0.19).

Contextual messages also had an impact on probabilistic discounting [*F*_(4, 178)_ = 3.26, *p* < 0.05]. Compared with participants in the long-time and low-probability conditions, participants in the short-time, and high-probability conditions more strongly discounted the value of 750 RMB received with a probability of 72% (t_ST−LT_ = −2.19, *p* < 0.05; t_ST−LP_ = −2.81, *p* < 0.01; t_HP−LT_ = −2.23, *p* < 0.05; t_HP−LP_ = −2.84, *p* < 0.01). No other differences were found (*p*s > 0.12).

#### Mediation effect of psychological distance

**Figure 2:**
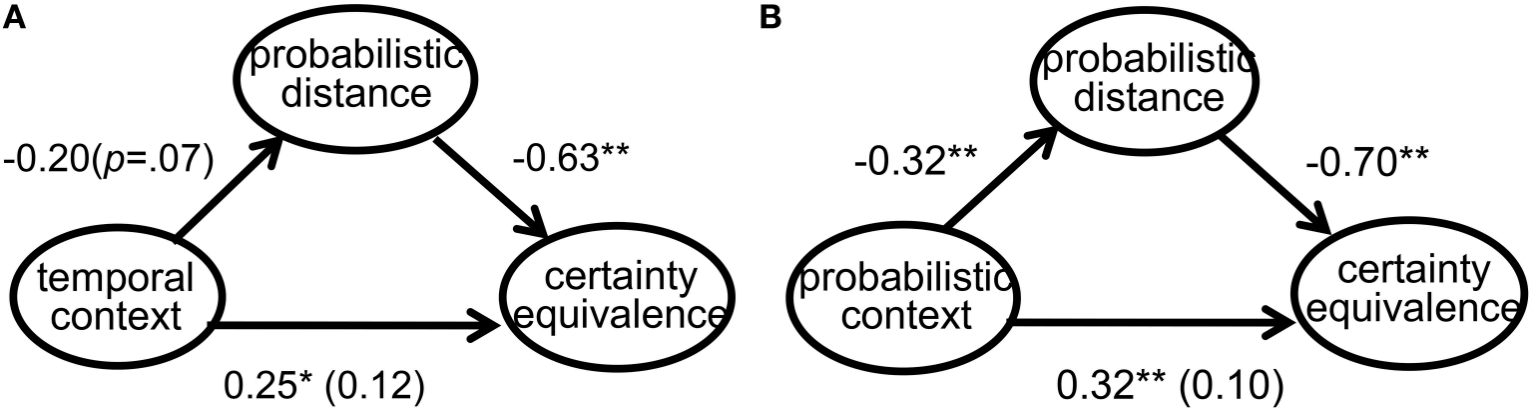
**(A)** Mediation analysis of probabilistic distance between temporal contexts and certainty equivalence estimation. **(B)** Mediation analysis of probabilistic distance between probabilistic contexts and certainty equivalence estimation.

A mediation analysis illustrated that probabilistic distance estimation mediated the relationships between types of probability-related contextual messages and certainty equivalence assessment and between types of time-related contextual messages and certainty equivalence assessment. Figures 2A,B show the details of these regression results. Both messages involving time and uncertainty influenced certainty equivalence estimation through the process of probabilistic distance formation.

## Discussion

Based on DbS theory and psychological distance theory, we reasoned that contextual messages related to time or uncertainty will influence both the perception of time and probability and the degree of value discounting. The current research supported this assumption. In particular, Study 1 revealed that participants memorizing long-time and low-probability events treated certain delay as more proximal and exhibited a less degree of temporal discounting than those memorizing short-time and high-probability events. The comparable effect of contextual messages on estimations of probabilistic distance and certainty equivalence was manifested in Study 2. Therefore, the current paper answered the question of “whether and why information pertaining to certain time or probability will influence our assessment of delayed or uncertain outcomes.”

### Implications for temporal and probabilistic discounting

The present study can be regarded as an example of the priming effect within time and probability decisions. Several studies have found that various activated concepts or schemas influence value discounting. For instance, in the domain of inter-temporal decision making, Rounding et al. (2012) tested the effects of implicit religious primes on self-control and found that when religious themes were made implicitly salient, people exercised greater self-control in delaying gratification. Israel et al. (2014) demonstrated that the priming of vacation scenes increased individuals' present preference, whereas pictures of older people enhanced people's patience with future. Similarly, with respect to decision making under uncertainty, it has been found that preferences regarding risky rewards are affected by priming stimuli such as experiences of past wins (Ludvig et al., 2015), incidental reward cues (Knutson et al., 2008), or specific colors (Kliger and Gilad, 2012). Consistent with previous research on the priming effect, the current study showed that activation of knowledge regarding time and uncertainty—such as the self-updating cycle of human cells and the success rate of iris certification systems—can alter our financial decision making as well.

Our study is also in line with the DbS theory (Stewart et al., 2006). This theory argues that people's judgments and preferences are constructive and shaped by sample items from both the immediate context and memory. Since both crucial components of this theory—sampling and comparison—were illustrated in the current study thus, the current findings supported the notion of DbS.

### Implications for psychological distance theory

One principal assumption of psychological distance theory is that temporal, probabilistic, social, and spatial distances have a common meaning and converge around one unitary construct—psychological distance (Trope and Liberman, 2010). The majority of studies testing this hypothesis have focused on how one type of psychological distance of a target influences individuals' perceptions of that target along other distance dimensions (Stephan et al., 2011; Fiedler et al., 2012; Wakslak, 2012; Maglio et al., 2013; Yan, 2014). For instance, Fiedler et al. (2012) asked participants to imagine themselves engaging in a specific social behavior and to indicate the psychological distance of that particular event along four psychological dimensions. The results indicated significantly positive correlations among all four distance estimations. Recently, Yan (2014) completed a series of studies thoroughly examining the “distance on distance effect.” He found that sitting closer to a prize increased one's perceived likelihood of winning, that London was perceived as closer as the Olympics approached, that events in distant places were viewed as relatively uncertain and that Western surnames increased estimations of the amount of time needed to put thoughts into action. Of note, in these studies, all divergent dimensions of psychological distance pointed to a common target.

The present study differs from previous research in that it explored the effect of irrelevant messages related to time and uncertainty on temporal and probabilistic distance estimation and value discounting. It showed that compared with those receiving short-time or high-probability messages, participants receiving long-time or low-probability messages viewed certain delay and uncertainty as mentally closer and exhibited a less degree of value discounting. As information related to time or probability can affect our performance in terms of both inter-temporal and risk-related decisions, the notion that time and uncertainty are similar in nature is supported.

In particular, why does the experience of one type of long distance increase the subjective magnitude of another type of distance when they belong to the same target but decrease the sense of remoteness when they are unrelated? This effect can be explained by Schwarz and Bless' inclusion/exclusion model (1992, 2007). This theory holds that when people evaluate a target with a reference, they first compare the target and the reference. If the target and reference belong to the same category, according to standards such as similarity and closeness, the likelihood of assimilation increases; if they do not belong to the same category, contrast dominates. Because the time and probability within priming messages were distinct from those within decision tasks, the occurrence of the contrast effect was reasonable in the current setting.

### Implications for application

It is well established that one's academic performance is closely associated with her or his self-control and patience (e.g., Tangney et al., 2004; Duckworth and Seligman, 2005). For instance, in Duckworth and Seligman' study (Duckworth and Seligman, 2005), students' self-discipline significantly predicted their final grades and standardized achievement-test scores, even when controlling for the impact of IQ. Nonetheless, the current study demonstrates that the content of knowledge may also affect one's self control capacity. While messages regarding longtime and low likelihood will give rise to a smaller trend of value discounting, messages related to short duration or high probability have the opposite effect. Therefore, the type of knowledge people acquire will shape their performance within inter-temporal and risky decision field.

Another topic related to the current study is persuasion and attitude change. Because our judgments and preferences are influenced by contextual messages, various strategies can be utilized to encourage or strengthen long-term-oriented behaviors. For instance, Hershfield et al. (2014) found that when the United States was framed as an old country rather than a young country, participants donated more money to an environmental organization. Similarly, our study suggests that when confronted with information emphasizing a long-time or a low-probability event, people will perceive a given delayed or uncertain outcome as more mentally adjacent and consider it more seriously.

### Limitations and future directions

The present study has some limitations. First, only one value of temporal delay (3 months) and one value of probability (72%) were adopted in the decision tasks. Because temporal delay and uncertainty largely vary within daily lives, whether our results can be generalized to other time and probability conditions remains to be tested. Second, several important issues concerning temporal and probabilistic discounting—such as the magnitude of rewards and the valence of outcomes—were not examined. Given the particular roles of these variables in decision making, it is necessary to explore the contextual effect on value discounting with differing levels of rewards and losses. Third, of note, the units of time in the long-time and short-time contextual messages and in the temporal decision task were divergent (“year” in long-time context, “day” in short-time context, and “month” in temporal decision task). As several studies have revealed that units of measurement influence judgment and decision making (Monga and Rajesh, 2012), studies investigating the current effect with identical units are needed.

Some directions for future research are suggested by our study. First, Study 1 and 2 indicated that contextual messages related to time or uncertainty shape both our temporal and probabilistic discounting. Because psychological distance theory assumes that temporal, probabilistic, social, and spatial distances are tied to one underlying construct, the present research might be expanded to social and spatial domains. Second, the selection of time and probability in the contextual messages was relatively arbitrary in the present study. The temporal duration was approximately half a century in the long-time condition and several days in the short-time condition, while uncertainty was ~999%0 in the high-probability condition and 1%0 in the low-probability condition. As the scope of time and uncertainty vary dramatically across domains—for instance, ranging from milliseconds in chemical reactions to billions of years in the lifespan of the universe—whether the effect observed in this study would be strengthened or weakened by altering the scopes of time and uncertainty remains to be determined.

## Conclusion

The current studies investigated the effect of contextual messages on temporal and probabilistic discounting. The results indicated that compared with those who memorized short-term or high-probability events, participants who memorized long-term or low-probability events rated certain delay or uncertainty as mentally closer and exhibited a less degree of value discounting. In addition, the sense of distance from present or reality mediated the effect of contextual messages on value discounting.

## Author contributions

All authors listed, have made substantial, direct and intellectual contribution to the work, and approved it for publication.

## Funding

This research was supported by the National Natural Science Foundation of China (No. 71271189).

### Conflict of interest statement

The authors declare that the research was conducted in the absence of any commercial or financial relationships that could be construed as a potential conflict of interest.

## Footnotes

^1^In a pilot study, participants were asked to demonstrate their time or probability perceptions regarding the content of passages under each condition on a 7-point scale (“1”represented very short in time conditions and very likely in probability conditions, while “7”representedvery long in time conditions and very unlikely in probability conditions). The familiarity, memorization difficulty and incurred emotions toward each message were assessed on 7-point scales as well. Half of the participants appraised messages in long-time and high-probability conditions, and the remainder evaluated messages in short-time and low-probability conditions. The results indicated that the temporal duration under the long-time condition was judged as longer than that under the short-time condition [*t*_(70)_ = 6.24, p < 0.01] and that the likelihood in the low-probability condition was rated as smaller than that in the high-probability condition[*t*_(66)_ = 9.72, p < 0.01). In addition, these massages did not differ in terms of familiarity, memorization difficulty or accompanying emotion (ps > 0.23). Therefore, the effect of these messages could be attributed to their content with regard to time and uncertainty but not familiarity, memorization difficulty or evoked emotions.

## Appendix: the materials in the memory task

### Long-time messages

A hundred-year orchid is a type of orchid that has a long life span relative to that of other herbaceous plants. This plant can live and reproduce in harsh conditions with little water. The average life span of a hundred-year orchid is 50.6 years.

Human cells continuously self-update to maintain bodily health. The rate of renewal varies widely across cells, which have different structures and functions. The self-updating rate of cardiacstemcells is relatively long, i.e., ~32.5 years.

A half-life is the time needed for a radioactive atom to decay to half its initial volume. This index thus reflects the degree of stability of a given atom. The half-life of Caesium-137 is ~40.2 years, which is relatively long for known isotopes.

### Short-time messages

The short-lived chrysanthemum is a type of chrysanthemum that has a short life span relative to that of other herbaceous plants. This plant can live and reproduce under harsh conditions with little water. The average life span of a short-lived chrysanthemum is 2.5 days.

Human cells continuously self-update to maintain bodily health. The rate of renewal varies widely across cells, which have different structures and functions. The self-updating rate of intestinalepithelialcells is relatively short, i.e., ~3.2 days.

A half-life is the time needed for a radioactive atom to decay to half its initial volume. This index thus reflects the degree of stability of a given atom. The half-life of Caesium-67 is ~1.6 days, which is relatively short for known isotopes.

### Low-probability messages

An organism's DNA is quite stable and rarely mutates during duplication. It is estimated that the duplication variation rate of DNA is < 0.1 thousandths. That is, out of 10,000 duplications, replicated DNA will differ from its parent generation only once.

Competition in the publishing industry is intense. It is estimated that the probability of a book becoming a bestseller after publication is ~2.1 thousandths. That is, among 480 books, only one will achieve sufficient attention and acquire a large readership.

Iris certification is an advanced technology used in identity recognition. It is estimated that the failure rate of this system is less than 0.2 thousandths. That is, out of 5400 recognition tests, only one detection error occurs.

### High-probability messages

An organism's DNA is quite stable and rarely mutates during duplication. It is estimated that the duplication accuracy rate of DNA is 999.9 thousandths. That is, out of 10,000 duplications, replicated DNA will be fully identical to its parent generation 9999 times.

Competition in the publishing industry is intense. It is estimated that the probability of a book achieving little response among readers after publication is ~997.8 thousandths. That is, among 480 books, 479 of them will receive little attention and be forgotten quickly.

Iris certification is an advanced technology used in identity recognition. It is estimated that the success rate of this system is 999.8 thousandths. That is, out of 5400 times recognition tests, detection will be correct 5399 times.
